# DrugCentral 2021 supports drug discovery and repositioning

**DOI:** 10.1093/nar/gkaa997

**Published:** 2020-11-05

**Authors:** Sorin Avram, Cristian G Bologa, Jayme Holmes, Giovanni Bocci, Thomas B Wilson, Dac-Trung Nguyen, Ramona Curpan, Liliana Halip, Alina Bora, Jeremy J Yang, Jeffrey Knockel, Suman Sirimulla, Oleg Ursu, Tudor I Oprea

**Affiliations:** Department of Computational Chemistry, “Coriolan Dragulescu’’ Institute of Chemistry, 24 Mihai Viteazu Blvd, Timişoara, Timiş, 300223, România; Translational Informatics Division, Department of Internal Medicine, University of New Mexico Health Sciences Center, Albuquerque, NM 87131, USA; UNM Comprehensive Cancer Center, University of New Mexico Health Sciences Center, Albuquerque, NM 87131, USA; Translational Informatics Division, Department of Internal Medicine, University of New Mexico Health Sciences Center, Albuquerque, NM 87131, USA; Translational Informatics Division, Department of Internal Medicine, University of New Mexico Health Sciences Center, Albuquerque, NM 87131, USA; College of Pharmacy, University of New Mexico Health Sciences Center, Albuquerque, NM 87131, USA; National Center for Advancing Translational Science, 9800 Medical Center Drive, Rockville, MD 20850, USA; Department of Computational Chemistry, “Coriolan Dragulescu’’ Institute of Chemistry, 24 Mihai Viteazu Blvd, Timişoara, Timiş, 300223, România; Department of Computational Chemistry, “Coriolan Dragulescu’’ Institute of Chemistry, 24 Mihai Viteazu Blvd, Timişoara, Timiş, 300223, România; Department of Computational Chemistry, “Coriolan Dragulescu’’ Institute of Chemistry, 24 Mihai Viteazu Blvd, Timişoara, Timiş, 300223, România; Translational Informatics Division, Department of Internal Medicine, University of New Mexico Health Sciences Center, Albuquerque, NM 87131, USA; Department of Computer Science, University of New Mexico, Albuquerque, NM 87131, USA; Department of Pharmaceutical Sciences, School of Pharmacy, The University of Texas at El Paso, TX 79902, USA; Computational and Structural Chemistry, Merck & Co., Inc., 2000 Galloping Hill Road, Kenilworth, NJ 07033, USA; Translational Informatics Division, Department of Internal Medicine, University of New Mexico Health Sciences Center, Albuquerque, NM 87131, USA; Computational and Structural Chemistry, Merck & Co., Inc., 2000 Galloping Hill Road, Kenilworth, NJ 07033, USA; Department of Rheumatology and Inflammation Research, Institute of Medicine, Sahlgrenska Academy at University of Gothenburg, 40530 Gothenburg, Sweden; Novo Nordisk Foundation Center for Protein Research, Faculty of Health and Medical Sciences, University of Copenhagen, 2200 Copenhagen, Denmark

## Abstract

DrugCentral is a public resource (http://drugcentral.org) that serves the scientific community by providing up-to-date drug information, as described in previous papers. The current release includes 109 newly approved (October 2018 through March 2020) active pharmaceutical ingredients in the US, Europe, Japan and other countries; and two molecular entities (e.g. mefuparib) of interest for COVID19. New additions include a set of pharmacokinetic properties for ∼1000 drugs, and a sex-based separation of side effects, processed from FAERS (FDA Adverse Event Reporting System); as well as a drug repositioning prioritization scheme based on the market availability and intellectual property rights forFDA approved drugs. In the context of the COVID19 pandemic, we also incorporated REDIAL-2020, a machine learning platform that estimates anti-SARS-CoV-2 activities, as well as the ‘drugs in news’ feature offers a brief enumeration of the most interesting drugs at the present moment. The full database dump and data files are available for download from the DrugCentral web portal.

## INTRODUCTION

DrugCentral integrates a broad spectrum of drug resources related to chemical structures, biological activities, regulatory data, pharmacology and drug formulations (1). Since 2018, DrugCentral has continuously strengthened its role as a key resource for the worldwide scientific community being additionally cross-referenced by several resources, such as UniProt (2), ChEBI (3), Hetionet (4), GUILDify (5), UniChem (6) and Guide to Pharmacology (7). DrugCentral served as primary resource for RepoDB, a drug repurposing database (8), a time-resolved computational drug repurposing algorithm (9), and an adverse drug event network for computational toxicology predictions (10). First introduced and published in the 2017 NAR database issue (1), DrugCentral reconciles the basic scientist's understanding of the ‘drug’ concept (active pharmaceutical ingredient) with the view of the patient and healthcare practitioner (pharmaceutical formulation). Since its initial launch, the two DrugCentral papers (1,11) were cited more than 160 times cf. Google Scholar, and the website is accessed on average by ∼8000 visitors monthly, with a monthly average of ∼20 000 page views and over 20 000 full database downloads per year (as of 15 September 2020). Throughout regulatory and scientific documents, several terms are often used interchangeably: drug substance, new chemical (or molecular) entity and active (pharmaceutical) ingredient. While these terms have precise contextual meaning, in this paper preference is given to the term ‘drug’ as synonymous with these three concepts. The term ‘formulation’ is used when discussing pharmaceutical products.

The current update adds newly approved drugs by the US Food and Drug Administration (FDA, https://www.fda.gov/home) and the European Medicines Agency (EMA, https://www.ema.europa.eu/en) up to 31 March 2020. Drugs approved by Japan Pharmaceuticals and Medical Devices Agency (PMDA, https://www.pmda.go.jp/english/index.html) were also monitored up to the latest information available, i.e. November 2019. In addition, for numerous drugs present in DrugCentral since 2018, regulatory agency information was added according to their approval status.

An important component of drug discovery and repositioning is information related to the pharmacokinetic (PK) properties of drugs, e.g. maximum recommended dose or half-life, as well as information related to side effects. In this regard, DrugCentral 2021 introduces critically reviewed information on PK, thus increasing the clinical pharmacology-related information coverage for drugs. Furthermore, adverse drug events separated by sex are tabulated at the drug level, to increase our understanding of drug safety.

Sudden outbreaks can rapidly impact global health, as evidenced by the COVID-19 pandemic, caused by severe acute respiratory syndrome coronavirus 2 (SARS-CoV-2). This pandemic has accelerated the need to rely on computational platforms (12) capable of identifying and advancing novel therapeutics for clinical evaluation. In this regard, the current DrugCentral update enables computational and medicinal chemists with (i) drug repositioning categories, i.e. an in-depth classification of drugs based on current market status and intellectual property rights in the US (13), to prioritize new therapeutic uses for ‘old drugs’; and (ii) a suite of machine learning models that predict anti-SARS-CoV-2 activities, REDIAL-2020 (14), to prioritize compounds against COVID-19.

## CURRENT CONTENT

### Active pharmaceutical ingredients

The current DrugCentral update includes 109 newly approved drugs and two molecules (mefuparib and EIDD-2801, or ) with anti-SARS-CoV-2 potential to the 4531 indexed in 2018 (11). The vast majority of these were approved by the US FDA (95 drugs), followed by EMA (36 drugs), with 31 overlapping drugs. Compared to the additions in 2018, the number of newly approved drugs in Japan has nearly tripled, i.e. 16 new drugs compared to 6. In the past two years, the ratio of newly approved drugs between small organic molecules and biologics has changed in favor of the first class (70 small molecule drugs compared to 35 biologics), which contrasts with a more balanced ratio encountered in the last version of the database (11). Compared to the 2018 update, we note increases in the number of approved subtypes of biologics, such as antibody-drug conjugates (60% increase), oligonucleotides (50% increase) and monoclonal antibodies (30% increase). Approximately, half of the drugs processed (i.e. 52) are orphan drugs (15) pointing out the therapeutic gain in the group of rare diseases (15,16). Out of the newly added drugs, the ChEMBL database (17) indexes 104 (91) of the 111 drugs, KEGG (18) indexes 107, DrugBank captures 105 and the Guide to Pharmacology 77 drugs, respectively (Table 1).

**Table 1.** Differences in data content between DrugCentral 2016 (first release), 2018 and 2021 (current release)

Entities (annotated drugs, or active pharmaceutical ingredients)			
	DrugCentral 2016	DrugCentral 2018	DrugCentral 2021
*Active pharmaceutical ingredients*	4444	4531	4642
FDA drugs	2021	2094	2220
EMA drugs	239	272	354
PMDA drugs	80	86	167
Small molecules	3799	3825	3876
Biologics and peptides	239	282	315
Other drugs	294	309	395
Parent molecules	199 (308)	211 (327)	216 (332)
*Drug efficacy targets*	837 (1689)	855 (1756)	872 (1760)
Human protein targets	600 (1387)	613 (1447)	659 (1534)
Infectious agents targets	194 (221)	197 (224)	212 (230)
Protein–drug crystal complex (PDB)	48 (82)	333 (139)	411 (165)
All protein–drug crystal complex (PDB)	1452 (283)	3991 (433)	5576 (799)
Bioactivity data points	13 825 (1792)	15 481 (1911)	16 843 (2052)
Human proteins	10 427 (1605)	11 241 (1692)	12 373 (1837)
Other species	3398 (1002)	4240 (1175)	4470 (1235)
*Pharmacological classification*			
WHO ATC code	4195 (2941)	4889 (2978)	5067 (3082)
FDA Established Pharmacologic Class	428 (1165)	450 (1220)	462 (1256)
MeSH pharmacological action	424 (2529)	457 (2615)	447 (2661)
ChEBI ontology roles	285 (1487)	295 (1529)	303 (1607)
*Drug indications*	2224 (2247)	2167 (2371)	2241 (2496)
Drug contra-indications	1458 (1376)	1407 (1379)	1415 (1399)
Drug off-label uses	847 (646)	817 (641)	818 (654)
*Pharmaceutical products*	67 064 (1660)	77 484 (1716)	108 035 (1810)
Rx pharmaceutical products	29 665 (1561)	34 192 (1609)	56 515 (1697)
OTC pharmaceutical products	37 399 (286)	43 292 (296)	51 520 (319)
*External identifiers*	61 349 (4444)	69 516 (4531)	63 658 (4639)
CAS registry number	6072 (4444)	6200 (4531)	6350 (4642)
PubChem Compound Id	4175 (4175)	4289 (4308)	4399 (4412)
FDA Unique Ingredient Identifier (UNII)	4304 (4304)	4391 (4391)	4505 (4505)
ChEMBL-db id	5615 (4075)	6077 (4330)	6473 (4469)
WHO INN id	3519 (3519)	3589 (3589)	3700 (3700)
SNOMED-CT	4745 (2637)	4968 (2815)	5193 (2910)
KEGG DRUG	3501 (3501)	3576 (3576)	3697 (3698)
NDFRT	4171 (2406)	4256 (2479)	3464 (3314)
RxNorm RxCUI	2897 (2897)	2988 (2991)	3107 (3110)
IUPHAR/BPS ligand id	1345 (1345)	1391 (1395)	1599 (1599)
UMLS CUI	2839 (2839)	2835 (2835)	2835 (2835)
CHEBI	2557 (2557)	3824 (3830)	3855 (3861)
MeSH	4063 (3846)	4180 (3946)	4299 (4056)
DrugBank	2473 (2388)	2773 (2858)	3685 (3699)
Protein Databank ligand id	646 (618)	713 (695)	695 (659)

### Bioactivity data and mechanism of action

The present release adds 1379 new bioactivity datapoints from ChEMBL (17) and the Guide to Pharmacology (7) using automated pipelines, 79% and 8%, respectively; and manually curated scientific literature and approved drug label data (13%). Newly introduced drugs are associated with 551 bioactivity points from ChEMBL (65.5%), manual curation from literature (24.14%), the Guide to Pharmacology (6.17%) and approved drug labels (4.17%), respectively; as well as 109 mechanism of action (MoA or **Tclin** proteins—*vide infra*) targets, with kinases (26%) and enzymes (21%) representing the major target categories, followed by G-protein-coupled receptors—GPCRs (17%) and tumor-associated antigens (9%). Since 2018, 46 novel MoA targets, associated with 32 newly approved drugs, have been introduced (Table 2).

**Table 2.** New active pharmaceutical ingredients with novel mechanisms of action approved since the 2018 release of DrugCentral

Active Ingredient(s)	Target	Target Class^a^	Agency	Indication
crizanlizumab	SELP	Adhesion	FDA	Vaso-occlusive crisis in sickle cell disease
luspatercept	GDF11, MSTN	Cytokine	FDA	Beta thalassemia
emapalumab	IFNG	Cytokine	FDA	Primary hemophagocytic lymphohistiocytosis
prabotulinumtoxinA	SNAP25	Cytosolic other	FDA	Rhytidectomy of glabellar frown lines
botulinum toxin type A	SNAP25	Cytosolic other	EMA	Rhytidectomy of glabellar frown lines
andexanet alfa	rivaroxaban, apixaban	Drug	FDA, EMA	Direct-acting anticoagulant adverse reaction
roxadustat	EGLN1, EGLN2, EGLN3	Enzyme	PMDA	Anemia in chronic kidney disease, refractory anemia
ivosidenib	IDH1	Enzyme	FDA	Acute myeloid leukemia
romosozumab	SOST	Glycoprotein	FDA	Postmenopausal osteoporosis
fremanezumab	CALCA	GPCR	FDA, EMA	Migraine
galcanezumab	CALCA	GPCR	FDA	Migraine
erenumab	CALCRL	GPCR	FDA, EMA	Migraine
ubrogepant	CALCRL	GPCR	FDA	Migraine
lasmiditan	HTR1F	GPCR	FDA	Migraine
cannabidiol	GPR55	GPCR	FDA, EMA	Lennox-Gastaut syndrome, severe myoclonic epilepsy in infancy
bremelanotide	MC4R	GPCR	FDA	Lack or loss of sexual desire
larotrectinib	NTRK2, NTRK3	Kinase	FDA, EMA	Malignant neoplasm
entrectinib	NTRK2, NTRK3, ROS1	Kinase	FDA, PMDA	Reactive oxygen species 1 positive non-small cell lung cancer, solid neoplasm with neurotrophic receptor tyrosine kinase gene fusion
duvelisib	PIK3CG	Kinase	FDA	Chronic lymphoid leukemia, malignant lymphoma - small lymphocytic, follicular non-Hodgkin's lymphoma
lorlatinib	ROS1, LTK, FER, FES, NTRK2, NTRK3, PTK2, PTK2B, TNK2	Kinase	FDA, EMA, PMDA	Non-small cell lung cancer lung cancer
fostamatinib	SYK	Kinase	FDA, EMA	Immune thrombocytopenia
ibalizumab	CD4	Membrane receptor	FDA, EMA	Human immunodeficiency virus infection
tagraxofusp	IL3RA	Membrane receptor	FDA	Blastic plasmacytoid dendritic cell neoplasm
selinexor	XPO1	Nuclear other	FDA	Relapse multiple myeloma
givosiran	ALAS1	RNA	FDA, EMA	Hepatic porphyria
volanesorsen	APOC3	RNA	EMA	Chylomicronemia syndrome
golodirsen	DMD	RNA	FDA	Duchenne muscular dystrophy
burosumab	FGF23	Secreted	FDA, EMA, PMDA	Familial x-linked hypophosphatemic vitamin D refractory rickets
voxelotor	HBA1	Transporter	FDA	Sickle cell disease
sotagliflozin	SLC5A1	Transporter	EMA	Diabetes mellitus type 1
tenapanor	SLC9A3	Transporter	FDA	Irritable bowel syndrome characterized by constipation
polatuzumab vedotin	CD79B	Tumor-associated antigen	FDA, EMA	Diffuse large B-cell lymphoma refractory
enfortumab vedotin	NECTIN4	Tumor-associated antigen	FDA	Metastatic urothelial carcinoma
caplacizumab	VWF	Unclassified	EMA, FDA	Thrombotic thrombocytopenic purpura

^a^SELP, P-selectin; GDF11, Growth/differentiation factor 11; MSTN, Growth/differentiation factor 8; IFNG, Interferon gamma; SNAP25, Synaptosomal-associated protein 25; EGLN1, Egl nine homolog 1; EGLN2, Egl nine homolog 2; EGLN3, Egl nine homolog 3; IDH1, Isocitrate dehydrogenase [NADP] cytoplasmic; SOST, Sclerostin; CALCA, Calcitonin gene-related peptide 1; CALCRL, Calcitonin-gene-related peptide receptor; HTR1F, 5-hydroxytryptamine receptor 1F; GPR55, G-protein coupled receptor 55; MC4R, Melanocortin receptor 4; NTRK2, BDNF/NT-3 growth factors receptor; NTRK3, NT-3 growth factor receptor; ROS1, Proto-oncogene tyrosine-protein kinase ROS; PIK3CG, Phosphatidylinositol 4,5-bisphosphate 3-kinase catalytic subunit gamma isoform; LTK, Leukocyte tyrosine kinase receptor; FER, Tyrosine-protein kinase Fer; FES, Tyrosine-protein kinase Fes/Fps; PTK2, Focal adhesion kinase 1; PTK2B, Protein-tyrosine kinase 2-beta;TNK2,Activated CDC42 kinase 1; SYK, Tyrosine-protein kinase SYK; CD4,T-cell surface glycoprotein CD4; IL3RA, Interleukin-3 receptor; XPO1,Exportin-1; ALAS1, aminolevulinate synthase1 (ALAS1) mRNA; APOC3, apolipoprotein C-III (apoC-III) mRNA; DMD, exon 53 of dystrophin pre-mRNA; FGF23, Fibroblast growth factor 23; HBA1, hemoglobin subunit alpha; SLC5A1, Sodium/glucose cotransporter 1; SLC9A3, Sodium/hydrogen exchanger 3; CD79B, B-cell antigen receptor complex-associated protein beta chain; NECTIN4, Nectin-4; VWF, von Willebrand factor;

Our knowledge-based protein classification (19) bins human proteins into four categories, according to their ‘target development level’ (TDL): **Tclin** are MoA-designated drug targets via which approved drugs act (15,20,21), currently 659 human proteins; **Tchem** are proteins that are not **Tclin**, but are known to bind small molecules with high potency; **Tbio** includes proteins that have Gene Ontology (22) ‘leaf’ (lowest level) term annotations based on experimental evidence; or meet two of the following three conditions: A fractional publication count (23) above five, three or more Gene RIF, ‘Reference Into Function’ annotations (https://www.ncbi.nlm.nih.gov/gene/about-generif), or 50 or more commercial antibodies, as counted in the Antibodypedia portal (24). The fourth category, **Tdark**, currently includes ∼31% of the human proteome that were manually curated at the primary sequence level in UniProt, but do not meet any of the **Tclin**, **Tchem** or **Tbio** criteria. DrugCentral 2021 contains 669 **Tchem**, 219 **Tbio** and 14 **Tdark** proteins linked to 3859, 607 and 39 bioactivity points, respectively. These proteins are mapped onto the Target Central Resource Database (TCRD) and interfaced with the TCRD portal, Pharos, respectively (25,26).

### Pharmacological classification

New and existing drugs in DrugCentral were mapped (or remapped) into the latest versions of the World Health Organization Anatomic, Therapeutic and Chemical classification system (WHO ATC, https://www.whocc.no/), the FDA Established Pharmacologic Class (EPC, https://bit.ly/2OWiJdH), the Medical Subject Headings (MeSH) (27) and ChEBI (3) pharmacological classifications using the adaptive mapping schemes described in 2018. The resulting pharmacological additions are described in Table 1. Among novel drugs, 78 were linked to 136 pharmacologic classifications; 313 of the drugs were mapped to 424 additional pharmacologic terms.

### Pharmaceutical formulations

FDA pharmaceutical formulations were assessed using DailyMed (https://dailymed.nlm.nih.gov/) data, downloaded on 9 May 2020. A total of 31 731 new formulations with effective dates starting from 30 June 2018 were added to DrugCentral 2021. The vast majority of these products (82%) are for oral (17 052) and topical (9832) administrations. The percentage of human prescription (Rx) products (52.7%) remains only slightly higher compared to OTCs.

## NEW DATA AND FUNCTIONALITY

### Drug repurposing categories

**Figure 1.**
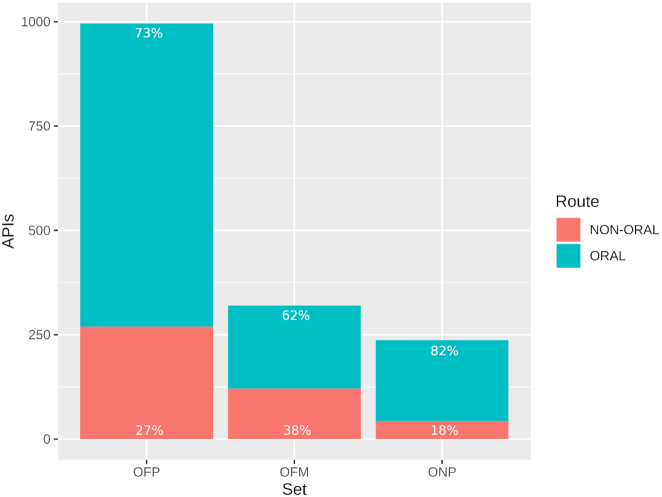
Bar plot showing the number of DrugCentral active pharmaceutical ingredients—drugs (formulated for oral versus non-oral route administration) mapped to FDA-OB and labeled as: OFP (on-market but off-patent), OFM (off-market) and ONP (on-market, on-patent), respectively (13).

The current version of DrugCentral includes a recently published drug repurposing categorization scheme (13), according to which drugs are sorted based on their market availability and intellectual property rights (including exclusivity protections) into three distinct categories: OFP, or off-patent, which are on-market drugs with expired patents or exclusivities; ONP, or on-patent, which are on-market drugs covered by current patents and exclusivity protections; and OFM, or off-market, which includes all previously marketed drugs that have been discontinued or withdrawn, respectively. The analysis, based on the US FDA’s Orange Book (FDA-OB), mapped small organic molecules and peptides from DrugCentral (having molecular weight between 100 and 1250) onto FDA-OB. In total, 996 drugs were categorized as OFP, 320 as OFM and 237 as ONP (Figure 1), respectively. These drugs can be found in a variety of pharmaceutical formulations, but oral drugs appear to be predominant in all three sets: 73% in OFP, 82% in ONP and 62% in OFM. Moreover, the data shows an increasing proportion of oral drugs in more recently approved drugs (i.e. ONP and OFP compared to OFM).

This classification scheme allows researchers to inform their decisions with respect to drug repositioning based on the existing intellectual property landscape. Given that, in time, novel drugs will be added and other drugs will change categories (i.e. ONP drugs naturally migrate to OFP and, possibly, to OFM), this drug repositioning classification will be updated on a yearly basis following the previously described workflow (13). This feature complements the pharmacopedic nature of DrugCentral, providing the scientific community (academia and industry) support to more efficiently advance ‘old’ drugs toward new therapeutic opportunities (28).

### ADMET-PK data

**Figure 2.**
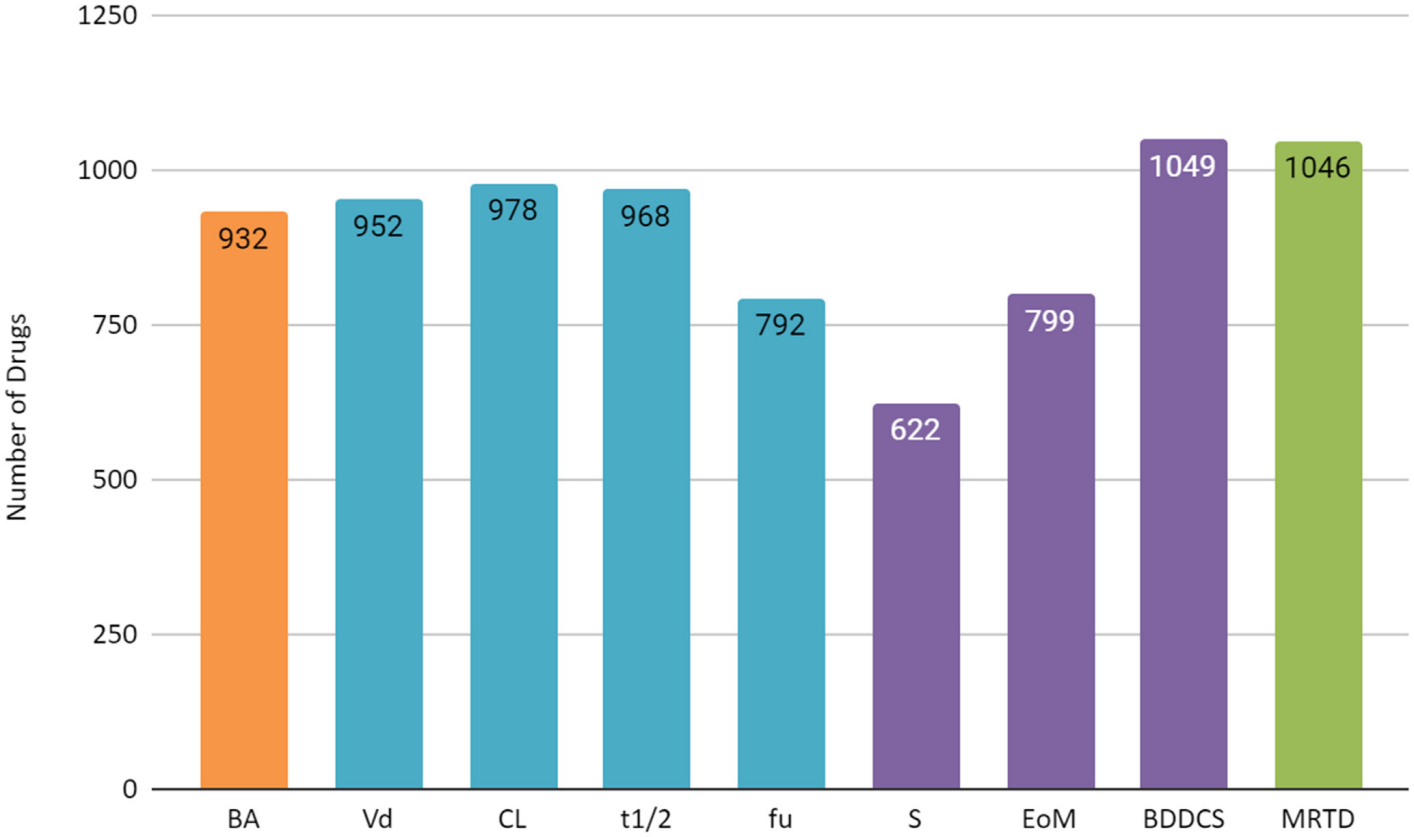
Bar plot showing the number of drugs indexed from literature sources for each ADMET-PK (absorption, distribution, metabolism, excretion and toxicity—pharmacokinetics) property. Colors indicate the different literature sources: orange for BA—bioavailability (29); blue for Vd—Volume of distribution, CL—Clearance, t1/2—half-life time and fu—fraction unbound (30); purple for S—water solubility, EoM—Extent of Metabolism and BDDCS—Biopharmaceutical Drug Disposition Classification System (31), (32); and green for MRTD—Maximum Recommended Therapeutic Daily Dose (33), respectively.

DrugCentral 2021 now includes nine measured properties that describe pharmacokinetics (PK) such as absorption, distribution, metabolism, excretion and toxicity (ADMET) for a number of drugs. These ADMET-PK data were retrieved from five authoritative references (29–33), which themselves are extensively curated compilations from biomedical literature or drug records. These ADMET-PK properties are highly relevant for understanding the fate of drugs in the human body, for estimating dosage regimens and for conducting data analyses or machine learning studies. The number of drugs indexed with each property is summarized in Figure 2. What follows is a brief description of the ADMET-PK properties incorporated in DrugCentral 2021.

- The absolute oral bioavailability (BA) indicates the fraction of the orally dosed drug that is absorbed through the gut, undergoes first-pass metabolism (gut and liver) and reaches systemic circulation.
- The volume of distribution at steady state (Vd) is the theoretical volume (expressed in L/kg) necessary to contain the measured steady-state drug concentration in plasma.
- The systemic (or total) clearance (CL) is the volume of plasma from which a drug is completely removed from the body. It is expressed as mL/min/kg and it is the sum of the clearance of the drug by each organ: kidneys, liver, etc.
- Half-life (t1/2) is the time (expressed in hours) it takes for a drug to decrease to half of its maximum concentration in plasma.
- The fraction unbound (fu) is the fraction of drug that is not bound to plasma proteins.
- Water solubility (S) indicates the degree of a drug dissolving in water at neutral pH and 37°C.
- The extent of metabolism (EoM) is the fraction of the drug (API) excreted unchanged (mainly, in urine).
- The Biopharmaceutical Drug Disposition Classification System (BDDCS) is an adaptation of the FDA Biopharmaceutical Classification System for bioequivalence studies. In BDDCS, drugs are assigned to four categories in accordance with solubility and EoM cutoffs: Class 1 are high solubility, extensively metabolized drugs; Class 2 are low solubility, extensively metabolized drugs; Class 3 are high solubility, poorly metabolized drugs; and Class 4 are low solubility, poorly metabolized drugs, respectively. It should be noted that the solubility used for BDDCS is the one defined by FDA guidance: the solubility of the formulated active ingredient at its highest approved dose strength, in 250 mL of water, at 37°C, over the pH range 1–6.8 (https://www.fda.gov/media/70963/download). BDDCS has proven to be useful in understanding the role of drug transporters (34), in predicting the brain permeability of drugs (35) and in understanding the PK specificity of drug targets (36). BDDCS, S and EoM data gathered from two separate publications (31,32).
- The Maximum Recommended Therapeutic Daily Dose (MRTD) is the dose threshold above which a drug starts to manifest adverse reactions. Therefore, it is a measure of the toxicity potential of a drug. While the original publication (33) reported MRTD in mg/kg/day units, whereas DrugCentral 2021 uses μM/kg/day (i.e. the mg quantities were divided by the molecular weight of the specific active ingredient). MRTD values were re-normalized to an average body weight of 70 kg instead of the original 60 kg, although the ‘average 70 kg man’ concept needs re-evaluation (37).

As new data points become available, these will be added in DrugCentral.

### Sex-differences in adverse drug events

FAERS (FDA Adverse Event Reporting System, https://open.fda.gov/data/faers/) data were first incorporated in DrugCentral 2018 (11). Compared to the 2018 release, there was a 10% increase in unique drugs (from 2023 to 2220), which are associated with 12,098 unique MedDRA terms (i.e. adverse events—AEs; Medical Dictionary for Regulatory Activities, https://www.meddra.org/), resulting in 739 990 drug-AE combinations. The larger the log likelihood ratio LLR value (38) for an AE, the more likely the event occurred due to a drug, and significant signals can be encountered for AEs with LLRs larger than the calculated drug-specific threshold values (*t*). Statistically relevant signals for the LLR test yield 1618 unique drugs associated with 8185 unique AEs, for a total of 147 191 (20%) significant drug-AE combinations. The DrugCentral 2021 FAERS dataset supports sex-specific granularity for AEs. An overview of the sex differences described in Table 3 shows a larger number of AEs reported for women compared to men. Indeed, at LLR > 5*t, the number of API-AE pairs almost doubles in females. This phenomenon, first reported in the US using FAERS data (39), and independently confirmed in the Netherlands (40), shows that sex bias in medical treatment persists, ten years after it was first discussed (41). Creating an interface that highlights sex-differences in AEs may facilitate further analyses and may reveal essential drug actions to pave the way for truly personalized medicine (42).

**Table 3.** Summary of sex-specific adverse event data from FAERS, at different LLR levels

Categories	Number of API-AE pairs (unique drugs/unique AEs)	
	MALE	FEMALE
LLR > 0	403 993 (1824/9160)	467 048 (1.936 /9872)
LLR > 2*t	31 740 (968/3316)	50 282 (1124/4163)
LLR > 5*t	12 014 (737/1735)	20 845 (866/2397)

AE: adverse event; LLR: log likelihood ratio; t, LLR threshold.

### REDIAL-2020

DrugCentral 2021 incorporates a web server named ‘REDIAL-2020’ to efficiently estimate anti-SARS-CoV-2 activities from molecular structure (14). REDIAL-2020 hosts a suite of machine learning (ML) models that represent various experimental assays related to live virus infectivity (LVI), viral entry (VE) and virus replication (VR) process. It currently consists of six ML models that represent six assays using data from the NCATS (National Center for Advancing Translational Sciences) COVID19 portal (43). These assays are: the SARS-CoV-2 cytopathic effect, CPE (LVI) (44); Vero E6 host cell cytotoxicity (LVI counterscreen); Spike-ACE2 protein-protein interaction (AlphaLISA; VE) (45), TruHit (VE) counterscreen; angiotensin-converting enzyme 2 (ACE2; VE) inhibition; and 3C-like proteinase (3CL or Mpro; VR) inhibition (46). These models use chemical structures (or drug names; or PubChem CIDs) as input; a similarity search retrieves similar compounds in the NCATS dataset, and sorts them according to the Tanimoto similarity score. In addition to anti-SARS-CoV-2 activities, the top 10 most similar entries compared to the query molecule are displayed. Promising compounds are the ones that are (i) active in the CPE but inactive in cytotoxicity LVI models; (ii) active in the Spike-ACE2 (AlphaLISA) model and inactive in both the TruHit and ACE2 counterscreen VE models; or (iii) active in 3CL (VR) model; or any combination of the above. We are committed to update the current models periodically and build additional models to represent more assays as new data gets available in the literature.

Initially for each assay type, ML models based on each descriptor category (fingerprint, pharmacophore and physicochemical) were developed by employing 22 different ML algorithms from *scikit-learn* (47). The best performing model from each descriptor type was used to build consensus models. Finally, the best performing models according to their performance on the validation and test sets (15% of the initial set, each) were picked and implemented in the REDIAL-2020 prediction server. Against three different external sets, these models exhibited predictivity in the range of 60–75%. An in-depth discussion of the models, their training procedures, performance, external predictivity and implementation are discussed elsewhere (14). Based on the same concept as the L1000 gene perturbation profile similarity, which was implemented in DrugCentral 2018 (11), REDIAL-2020 serves a complementary need, i.e. the search for drugs effective against COVID-19, as opposed to the evidence-based (factual) DrugCentral system. Both aim to support the process of drug discovery and repositioning.

### Drugs in the news

**Figure 3.**
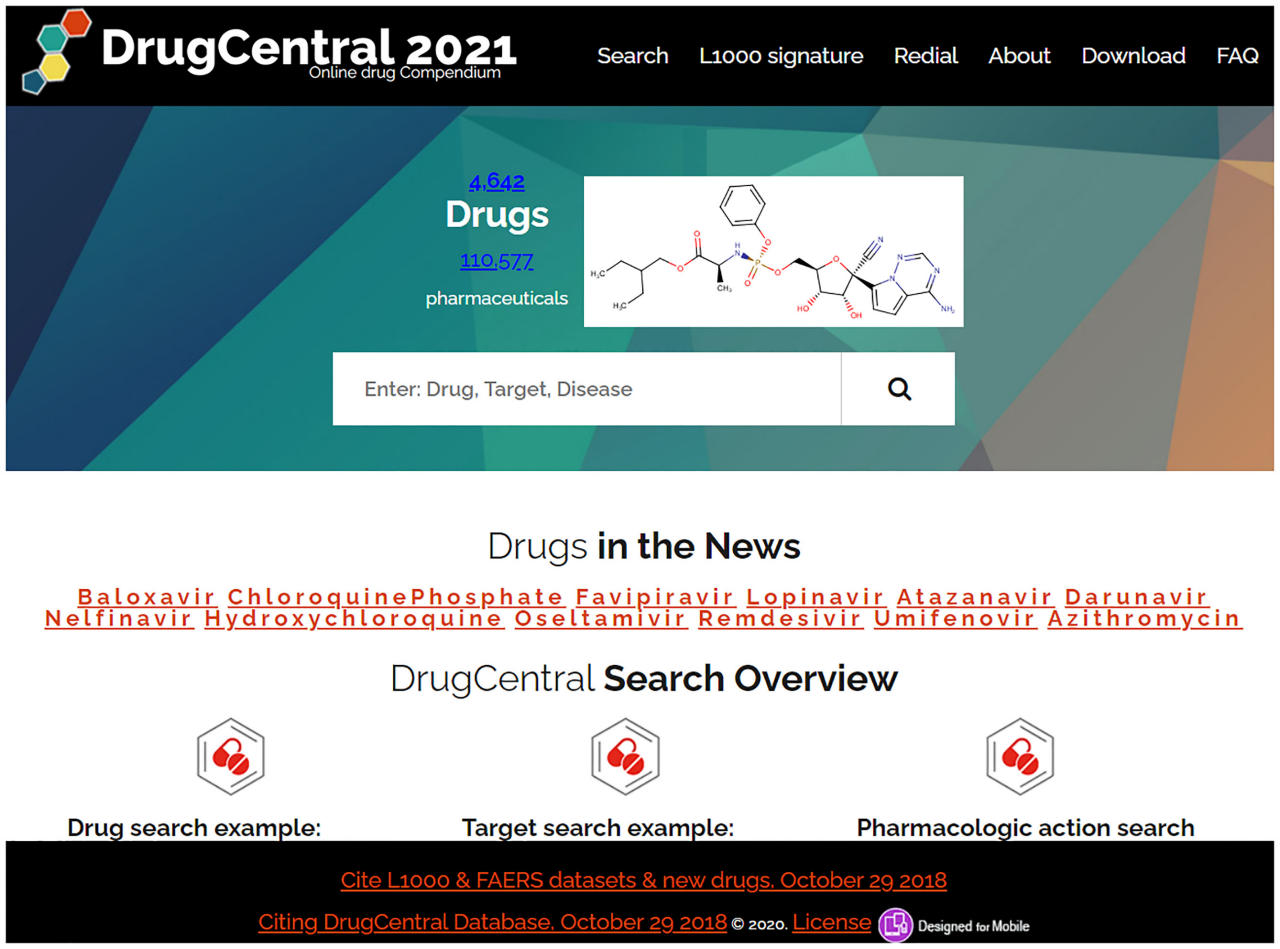
Front-page of the Drugcentral 2021 portal, featuring remdesivir in the chemical structure window.

Given the lack of approved therapeutic options, the COVID-19 pandemic has heightened the interest in approved medicines that are suitable for drug repositioning. A number of them have been used off-label in COVID-19 patients, and are therefore of interest to the community at large. Assessment of evidence for COVID-19-related treatments are frequently updated by the American Society of Health-System Pharmacists, AHSP (https://bit.ly/3mvXCQX). Reflecting heightened interest in COVID-19, the front-page of DrugCentral 2021 now includes a list of drugs that are ‘in the news’ (Figure 3). The current list includes favipiravir, which is not available in the US, but approved as Avigan in Japan and Russia and emergency approved in Italy (48) and remdesivir, which was granted emergency authorization in Japan and was FDA-approved as Veklury (https://bit.ly/33zA8Su), among other drugs.

## SUMMARY AND FUTURE DIRECTIONS

DrugCentral 2021 is up-to-date with drug marketing approvals and patent/exclusivity annotations up to 31 March 2020 and 23 June 2020, respectively. We incorporated ADMET-PK data and sex-based adverse events from FAERS, in addition to an anti-SARS-CoV-2 activities prediction server. At its core, DrugCentral continues to index {pharmaceutical formulation—drug—drug target—disease} association, although a significant number of additional attributes have been added to facilitate drug discovery and repositioning. We will continue to incorporate new drugs as soon as regulatory approvals are published. Drugs withdrawn due to other than safety reasons will be flagged in the OFM category, and all other drugs will be annually updated with respect to their marketing/patent/exclusivity status (13) in order to maintain easily accessible lists for drug repositioning. The FAERS interface will be streamlined to highlight sex differences in the drug safety profiles of existing drugs. Within the next six months, we plan to launch a chemical substructure and similarity search functionality. Last but not least, we have performed an extensive curation of veterinary drugs, which will be annotated in the next major DrugCentral release.

## DATA ACCESS

### Web interface

The DrugCentral web interface has been updated since the 2018 release to integrate novel data types and functionalities. The ‘Drugs in the news’ section will be updated monthly, by monitoring drugs that are widely associated with current events.

### Download

DrugCentral data can be downloaded in PostgreSQL format (full database dump available) for advanced data query, export and integration. User interaction with the local instance is facilitated through structured query language (SQL) examples as previously available, together with downloads of the chemical structures of the drugs in several formats (e.g. SDF, InChI and SMILES) and drugs bioactivity profiles in tabular format. The database is available via Docker container (https://dockr.ly/35G46a6), and public instance drugcentral:unmtid-dbs.net:5433. A Python API is also available (https://bit.ly/2RAHRtV).

## Supplementary Material

gkaa997_Supplemental_File
